# Factors Associated with Emergency Department Discharge After Falls in Residential Aged Care Facilities: A Rural Australian Observational Study

**DOI:** 10.3390/jcm14113893

**Published:** 2025-06-01

**Authors:** Gigi Guan, Geetha Ranmuthugala, Kadison Michel, Charlie Corke

**Affiliations:** 1 Department of Rural Health, Melbourne Medical School, The University of Melbourne, Shepparton 3630, VIC, Australia; geetha.ranmuthugala@unimelb.edu.au; 2 Critical Care Unit, Goulburn Valley Health, Shepparton 3630, VIC, Australia; kadison.michel1@gvhealth.org.au (K.M.); charlie.corke@gvhealth.org.au (C.C.); 3 School of Medicine, Deakin University, Geelong 3220, VIC, Australia

**Keywords:** advance care directives (ACDs), emergency department (ED), fall, residential aged care facilities (RACFs), nursing home, rural health services

## Abstract

**Background:** Falls are the leading cause of emergency department (ED) presentations among residential aged care facility (RACF) residents. This study identified the factors influencing the decision to discharge RACF residents from the ED following fall-related presentations. **Methods:** A single-centred, cross-sectional observational study was conducted in rural Shepparton, Victoria, Australia. The study included residents aged ≥65 in RACFs with fall-related ED presentations between 1 January and 19 November 2024. The main outcome was to determine factors that may prevent unwarranted ED transfers among RACF residents following falls. Statistical methods, including multivariate logistic regression, were used to examine factors associated with ED dispositions. **Results:** A total of 181 presentations (69.4%) were discharged, and 80 (30.6%) were admitted. The presence of an Advance Care Directive (ACD) (adjusted odds ratio [aOR] = 2.89; 95% confidence interval [CI]: 1.37–6.05) and lower triage levels (aOR = 2.69; 95% CI: 1.06–6.80) increased the odds of discharge. Major injuries (aOR = 0.20; 95% CI: 0.09–0.42) and obvious injuries (aOR = 0.24; 95% CI: 0.10–0.56) reduce discharge chances. Whether computed tomography brain scans were performed or anticoagulation therapy was used did not significantly influence ED discharge chances. **Conclusion:** In addition to traditional factors associated with ED discharge in post-fall patients from RACFs, an ACD was associated with increased discharge from the ED. Strengthening fall-specific advance care planning, improving ACD accessibility, and enhancing the clinical capacity of RACFs may reduce unnecessary ED transfers and better align care with residents’ goals, particularly in rural settings.

## 1. Introduction

Falls can lead to increased mortality and morbidity, particularly among older adults and residents of residential aged care facilities (RACFs). Notably, falls are the most common cause of emergency department (ED) presentations among RACF residents, with rates as high as 43% globally (95% confidence interval [CI]: 38–49%) [1]. According to the Australian Institute of Health and Welfare, 32.6% of RACF residents experienced at least one fall between April and June 2024 [2]. The number of RACF residents who have experienced a fall has remained consistent over the years, with 1.8% of falls resulting in significant injury [2]. Although the prevalence of significant injuries following falls is low, high fall rates contribute to substantial healthcare utilisation. A past study conducted in New South Wales, Australia, showed that fall-related ED presentations pose a significant economic burden to healthcare systems, particularly through an enormous number of presentations due to falls in RACFs [3]. Such costs to the healthcare system include ED transfers by ambulances, diagnostic imaging, hospital admissions, and high ED utilisation [3].

Although it is common for RACF residents to be transferred to the ED following falls, many transfers do not result in admission. A study conducted in Melbourne, Australia, reported that 71% of RACF residents’ fall presentations result in ED discharge [4]. This suggests that ED transfer decisions may not always align with clinical needs, with issues often exacerbated in rural and remote regions [5]. In these settings, geographic isolation, workforce shortages, and limited resources further increase reliance on ED transfers [6,7].

Despite the high rate of ED presentations among RACF residents following falls, little is known about the factors that influence the decision to discharge them from the ED. Previous research has mainly focused on fall prevention, injury characteristics, and systemic drivers of ED use [8]. Limited attention has been paid to clinical decision-making at the patient level, as studies have indicated that other factors may affect discharge decisions [9,10]. Factors such as advance care directives (ACDs) may be associated with a higher discharge rate in older adults from RACFs [10].

This study aimed to determine the factors that may help prevent unwarranted ED transfers among RACF residents following falls. Specifically, the study’s primary objective was to compare the clinical characteristics of RACF residents discharged from the ED and those admitted to the hospital following fall-related presentations. The secondary objectives were to evaluate the prevalence and impact of ACDs, ACD documentation, anticoagulation therapy before ED presentation, the proportion of computed tomography brain (CTB) scans and their findings, and other factors influencing discharge decisions.

## 2. Methods

### 2.1. Design and Setting

This study used a cross-sectional observational design, which offered a time-effective examination of patient demographics, management patterns, and disposition outcomes following ED presentations for falls. The study was conducted in a regional city hospital, Goulburn Valley Health (GVH). GVH is located in Shepparton, 180 km north of Melbourne, with a population of approximately 120,000 and a catchment with over 1100 residential aged care beds [11,12].

### 2.2. Data Source and Population

All data were obtained from GVH’s digital medical records (DMRs). From these records, we identified patients aged 65 or older who lived in RACFs and presented to the ED post-fall between 1 January and 19 November 2024. These presentations were identified using nursing triage notes, ICD-10 codes (Appendix A), and injury-related codes. We excluded presentations that had died on arrival and presentations related to mechanical or external causes, such as machinery, animals, road traffic accidents, or falls occurring outside RACFs.

### 2.3. Data Quality and Validation

A single author collected all the data in a spreadsheet, totalling 270 presentations. To ensure data accuracy, a second author randomly selected and reviewed 10% of the presentations (n = 27, 270 data entry points). A single discrepancy was identified and corrected, yielding an agreement rate of 99.6% in the inter-rater reliability test. This high rate demonstrated the accuracy of the dataset and a robust data extraction process with minimal risk of information bias.

### 2.4. Variables

The dependent variable was ED disposition, defined as whether the patient was admitted or discharged from the ED. In this study, multiple presentations for the same patient were indexed as individual presentations. However, we also recorded the number of multiple presentations. If patients were admitted to the short-stay unit (SSU), an observation unit located within the ED, they were considered continuously under ED care. The SSU or ED discharge outcomes determined the final admission status. Furthermore, patients transferred to other hospitals for the escalation of care were considered admissions.

The independent variables were age, sex, First Nations (Aboriginal or Torres Strait Islander) status, arrival method (ambulance or self-presentation), and keywords in triage notes, such as fall, pain, head strike, and obvious injuries (bony deformity, joint angulation, altered conscious state, haematoma, laceration). Other independent variables were the duration of ED stay, which was calculated in minutes from the time of arrival to the ED to the time of discharge. Referral before ED admission was categorised as ‘yes’ if the patient had been referred by a general practitioner (GP), nurse practitioner (NP), or the Victorian Virtual ED (VVED). It was categorised as ‘no’ if the patient had not undergone a prior medical review.

The presence and details of ACDs were recorded as either present (‘yes’) or absent (‘no’). Furthermore, we checked whether the ACD included preferences on transfer to the ED, cardiopulmonary resuscitation (CPR), or not for resuscitation (NFR). Anticoagulation therapy before ED admission was categorised as ‘yes’ (if the patient had used antiplatelets, anticoagulants, or both) or ‘no’. We also collected the triage level for each presentation; it ranged from 1 (most urgent) to 5 (least urgent) using the Australasian Triage Scale (ATS).

Further independent variables, including fall outcome, were classified according to injury severity as major (fracture, intracranial injuries, or multiple injuries) or minor (lacerations, sprains, or strains). The injury region was classified based on the anatomical region as either central (face, neck, or head) or peripheral (knee, ankle, or hand) (Appendix B and Appendix C). Patients who sustained both major and minor injuries were classified as major; similarly, patients who sustained both central and peripheral injuries were classified as having central injury. This approach aligns with clinical practice, reflection, and prioritisation, where central and severe injuries are typically the primary focus in emergency assessment and decision-making.

The prevalence and the results of CTB scans were classified as normal (no acute intracranial or intraparenchymal injury) or abnormal (acute intracranial bleeding or intraparenchymal injury).

We made the following assumptions during data analysis: if no documentation of an ACD was found, the patient was considered to have no ACD for the ED presentation. Abnormal CTB results were limited to acute intracranial or intraparenchymal injuries. Scalp haematoma and acute ischaemic stroke were considered normal classifications for analysis but were listed separately.

### 2.5. Data Analysis

Statistical analyses were performed using STATA SE 18.0, and statistical significance was set at α = 0.05. Descriptive statistics for continuous variables were presented using means, standard deviations (SDs), and ranges depending on the data distribution. The Shapiro–Wilk test was used to assess the normality of continuous variables. If normality was violated, the Mann–Whitney U-test was used for comparisons. Otherwise, the *t*-test was used. Categorical variables were summarised using frequencies and percentages. Inferential statistical tests were used to assess admission status. Specifically, chi-squared and Fisher’s exact tests were used to determine sex, injury severity, and CTB/ACD status between the discharged and admitted groups. Subgroup or sensitivity analyses were performed in available categories, such as CTB scans. As patients with triage level 1 were minimal (n = 1), this category was excluded from statistical testing.

Multivariate analysis was performed using logistic regression models to identify the factors affecting the decision to discharge RACF residents following fall-related ED presentations. The results were presented as odds ratios (ORs), 95% CIs, and two-tailed *p* values. Variables were retained in the final model based on a combination of statistical, clinical, and conceptual considerations. Particularly, variables with statistical significance (*p* < 0.05), a substantial effect (OR different from 1.0), variables that were identified as potential confounders, and clinically relevant factors were included. In this study, the presence of an ACD, the use of anticoagulation therapy, ATS level, and CTB scans were retained in the final model. Such a decision reflects the established clinically relevant current practice and policies that are supported by the existing literature. We aimed to improve model robustness, reflect real-world clinical reasoning, and ensure comparability with similar studies by including these variables. Furthermore, variables were excluded if they demonstrated high multicollinearity (variance inflation factor [VIF] > 10), perfect prediction, or a consistent lack of association across iterations (*p* > 0.20 and odds ratio across 1).

In this study, no missing data were noted during statistical analysis; therefore, a formal sample size calculation was not required. Only one presentation had missing data during data collection (the CTB result was unable to be retrieved). This presentation represented 1% of the data collected and was excluded from the presentations included. We assumed that the missing data occurred randomly and were thus unlikely to introduce systematic bias. A post hoc power analysis confirmed sufficient statistical power (*p* > 0.8) to detect clinically meaningful differences in admission status based on injury severity (Cohen’s h = 0.63) and the presence of ACD documentation (Cohen’s h = 0.39).

### 2.6. Ethics and Reporting

This study did not involve direct patient contact due to all data being sourced from DMRs. Consequently, obtaining informed consent was deemed impractical and unnecessary. Moreover, obtaining consent from each patient would have posed significant logistical challenges, introduced the risk of selection bias, and potentially compromised the study’s validity. This study was approved by the GVH Human Research Ethics Committee (approval number: GVH 25-24; 9 August 2024). The ethics committee approved the waiver of informed consent for this study. Further, this study was conducted according to the Strengthening the Reporting of Observational Studies in Epidemiology to ensure a rigorous study methodology (Appendix D). No external funding was received for the study.

## 3. Results

**Figure 1:**
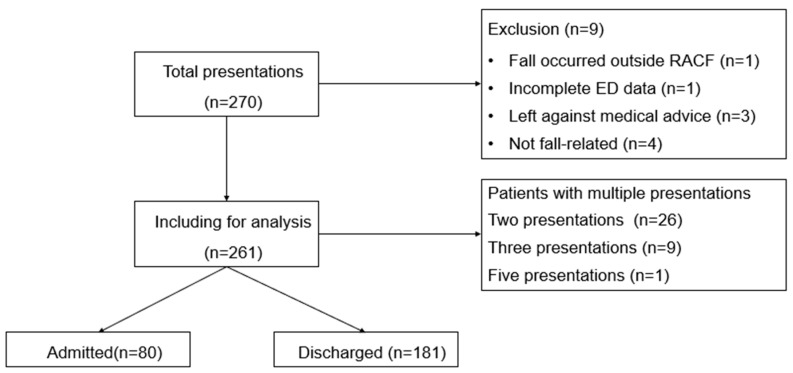
Flow chart of study sample selection.

During the study period, there were 270 presentations in total. We excluded three presentations because the patients left against medical advice, one presentation because the fall occurred outside an RACF, one presentation due to incomplete data, and four presentations because they were not fall-related. Consequently, the final sample contained 261 presentations. Regarding multiple presentations, 26 patients had two, 9 patients had three, and 1 had five presentations, respectively (Figure 1).

### 3.1. Discharge Rate and Patient Characteristics

Among the 261 presentations, 181 (69.4%) were discharged and 80 (30.6%) were admitted. The two groups did not differ based on age (Table 1; *p* = 0.538). A total of 50 presentations underwent medical review prior to transfer to the ED, with 30 reviewed by the VVED, 19 by GPs, and 1 by an NP (*p* = 0.428). Additionally, the two groups did not differ significantly based on sex (*p* = 0.974), First Nations status (*p* = 0.819), or arrival method (*p* = 0.359).

### 3.2. Factors Associated with ED Discharge

The duration of ED stay was significantly longer among admitted patients than among discharged patients (mean duration of 957 min [SD = 450.9] versus 485 min [SD = 368.2]; *p* < 0.001). After excluding ATS category 1 (n = 1), discharged patients showed lower triage levels than admitted patients (*p* = 0.028). Major injuries (*p* < 0.001) and central body (*p* < 0.001) injuries were associated with higher admission rates compared to the discharged group (Table 1).

### 3.3. Presence of ACDs and Their Details

The presence of an ACD significantly differed between the admitted and discharged groups, with Fisher’s exact *p* < 0.001. A total of 115 (44.1%) patients had an ACD in place at the time of ED presentation. Among these, 70 utilised the Victorian State ACD form, while the remaining used local RACF-developed ACD forms. In 10 further cases, the paramedics reported that an ACD existed at the RACF but that they were unable to be retrieved at the time of the ED presentation.

Of the 115 ACDs, 68 (59.0%) contained preferences for hospital transfer (15 ACDs explicitly advised against transfer to the ED). A total of 96 ACDs contained CPR preferences, with 85% expressing support for CPR attempts and 11 indicating NFR. In three cases, the ACD was not signed. In five cases, end-of-life or palliative care preferences were used instead of the ACD. Furthermore, two ACDs stated the patient’s values but did not contain specific medical instructions.

A narrative review of ACD documentation revealed the following patterns: Two nursing triage notes documented the presence of an ACD and the patient’s wishes, and four documented an NFR preference. Ten discharge summaries included the patients’ ACD wishes being completed by ED doctors. One presentation to the ED was at the request of a family member against the patient’s ACD wishes. Meanwhile, one ACD was found to contain inconsistent information regarding life-saving procedures.

### 3.4. CTB Scan and Anticoagulation Therapy Before ED Presentation

The use of anticoagulants, antiplatelets, or both was found in 156 (60.0%) cases. However, the two groups did not differ based on anticoagulation therapy before ED presentation (*p* = 0.746). Similarly, whether each presentation had a CTB scan was not significantly associated with admission (*p* = 0.140). CTB scan results showed abnormality in eleven cases, with three subdural haemorrhages, seven subarachnoid haemorrhages, and one intracerebral haemorrhage. None of these cases required neurosurgical intervention; all were managed conservatively or with palliative intent. The CTB findings also showed one chronic subdural haematoma, two scalp haematomas, and three ischaemic strokes. Thirteen patients underwent whole-body trauma imaging (pan scan); no intracranial injuries were identified in this group, and all patients were subsequently discharged.

### 3.5. Multivariate Analysis of Factors Associated with ED Discharge

**Figure 2:**
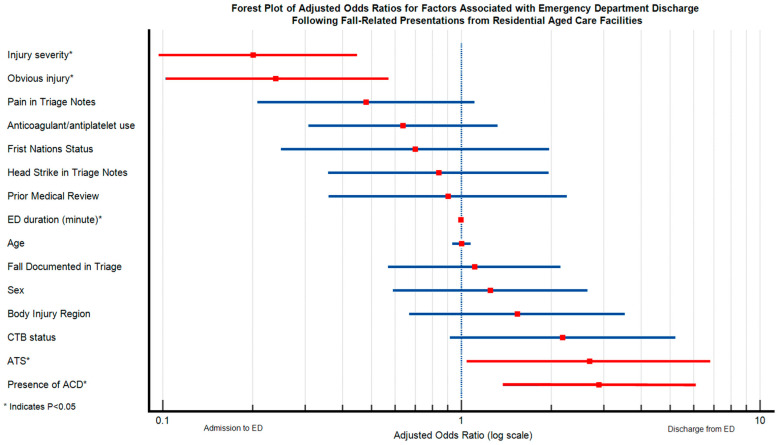
A Forest Plot of the adjusted odds ratios (aORs) and 95% confidence intervals (CIs) of factors associated with ED discharge. This forest plot displays adjusted odds ratios (aORs) with 95% confidence intervals for predictors of emergency department discharge (vs hospital admission) among residents of aged care facilities following a fall (n = 260). The adjusted model includes injury severity (major vs. minor), the presence of obvious injuries, the triage level using the Australian Triage Scale (level 1 to 5, with 1 being most urgent, 5 being least urgent), pain or head strike in triage notes documentation, whether a CTB was performed, anticoagulation use, and the presence of an ACD, among other covariates. aORs greater than 1 suggest increased odds of ED discharge, while aORs less than 1 suggest higher odds of hospital admission. The vertical reference line at OR = 1.0 indicates no association. The aORs and their 95% CIs in red indicate variables that did not cross the vertical reference line. Abbreviations: ACD = Advance Care Directive; CTB = computerised tomography brain scan; ATS = Australasian Triage Scale.

The final model comprised 260 presentations and demonstrated strong overall performance (LR χ² (15) = 113.21, *p* < 0.001; Pseudo-R² = 0.353; Figure 2).

After adjusting for covariates, the presence of an ACD significantly increased the odds of discharge (adjusted odds ratio [aOR] = 2.89; 95% CI: 1.37–6.05; *p* = 0.005). Minor injuries (aOR = 0.20; 95% CI: 0.09–0.42; *p* < 0.001), presentations without obvious injuries (aOR = 0.24; 95% CI: 0.10–0.56; *p* < 0.001), and lower triage levels (aOR = 2.69; 95% CI: 1.06–6.80; *p* = 0.037) were independently associated with increased chances of discharge. A longer duration of ED stay was inversely associated with discharge chances (aOR = 0.99 per minute; 95% CI: 0.99–0.99; *p* < 0.001). Other independent variables, such as whether CTB was performed, referral before presentation to the ED, and anticoagulation therapy before ED presentation, were not significantly associated with discharge chances in the adjusted model (Figure 2).

The Hosmer–Lemeshow test indicated adequate model calibration (χ² (8) = 11.72, *p* = 0.164), suggesting good agreement between the observed and predicted outcomes across risk deciles. A multicollinearity assessment demonstrated that all VIF values were below 1.4, with a mean VIF of 1.14, indicating acceptable independence among the predictor variables.

## 4. Discussion

### 4.1. Clinical Factors Associated with ED Discharge

This study identified key clinical and documentation-related factors that may affect the discharge of RACF residents following fall-related ED presentations. The presence of an ACD, injury severity, obvious injuries, duration of ED stay, and triage level affected discharge decisions. Other factors, such as CTB scans, anticoagulation therapy, and referral before ED presentation, did not influence discharge decisions.

While peripheral injuries, the absence of obvious injuries, and lower triage levels continue to justify discharge, non-clinical factors, particularly the presence of an ACD, may play a decisive role in ED disposition.

### 4.2. Advance Care Directives: Content, Access, and Clinical Impact

The National Framework for Advance Care Planning Documents supports scenario-based advance care planning [13]. However, despite falls being the leading cause of ED transfer from RACFs, none of the ACDs in this study included guidance specific to such events. This lack of detail creates uncertainty among RACF and ED staff members about whether the patient would prefer hospital transfers [14]. This gap highlights the need for scenario-based advance care planning, particularly for fall occurrences [15]. Furthermore, fall-related ED presentations strain emergency services and impose a significant cost on the healthcare system [3]. Strengthening end-of-life planning, especially through clear, scenario-based ACDs, may reduce avoidable ED presentations and hospitalisations [16]. In rural areas, enhanced training for RACF staff members and better integration with virtual emergency services could support timely, resident-centred care and reduce the systemic burden [17].

In this study, the presence of ACDs was significantly associated with increased ED discharge. However, this benefit hinges not only on the existence of an ACD but also on its accessibility and clarity. In this study, several ACDs were unavailable at the point of care or lacked fall-specific guidance, contributing to ED and RACF staff uncertainty. Given that falls are among the most frequent causes of ED transfer from RACFs, ACDs should routinely include scenario-specific instructions on fall management and ED transfer preferences. Doing so could reduce unnecessary presentations, align emergency care with residents’ goals, and support clinical decision-making in time-critical settings. Improved ACD access through shared digital platforms and staff training is critical in order to ensure that these documents can be meaningfully applied in real-world settings.

Even when ACDs are available and directive, adherence may be inconsistent. By law, valid ACDs should be followed; however, there is confusion among practitioners in practice [18]. Despite instructions not to transfer to the ED, the transfer might still occur if proxy decision-makers or family members overrule the ACD [18]. Our study confirmed this issue: we observed fifteen presentations where an ED transfer occurred despite the ACD advising against hospital transfer, including one transfer requested by a family member contrary to the ACD instructions. Healthcare professionals should understand state and local legislation regarding ACDs to respect patients’ wishes [18]. It is also important to educate and consult patients and proxy decision-makers [19]. A collaborative approach with patients and their family members is crucial to ensuring that patients’ wishes are followed and respected, as documented in their ACDs. Educating RACF staff (and family members) on respecting ACD instructions and managing falls within RACFs may reduce unnecessary transfers.

Our study highlights the need to increase ACD documentation and ensure their availability, particularly through scenario-based instructions such as *‘what to do after a fall’*. Such a practice would support RACF staff and reduce unnecessary escalations, especially in rural areas. Further research should examine how and what should be implemented to gain a better understanding of post-fall documentation in ACDs.

### 4.3. Capacity Constraints in RACFs and Systemic Factors

The decision to transfer post-fall patients to the ED is often multifactorial and beyond immediate clinical needs [20]. Many systemic factors influence this decision, such as the confidence of RACF nursing staff and facilities’ capacity to provide continuous post-fall monitoring, conduct proper medical assessments, and communicate effectively with family members [14].

RACF residents tend to have complex medical and social needs, as well as advanced frailty and comorbidities [21]. Thus, the onsite management of post-fall patients requires the nursing staff to have confidence in their skills [22]. When RACF staff feel uncertain about their ability to monitor or manage a patient’s condition, they are more likely to transfer the patient to the ED, even in clinically stable cases [23].

These challenges are further exacerbated in rural areas, where access to medical assessment and guidance is limited [5]. Without proper medical advice, RACF staff may resort to ED transfers to ensure patient safety [9,23]. Although telehealth consultations (such as via the VVED) are increasing, these services are not universally available or uniformly implemented across all facilities [24].

Addressing the capacity constraints within RACFs is fundamental to reducing unwarranted ED transfers and ensuring patient-centred care. These constraints include low confidence, a lack of access to clinical advice, and communication barriers. Moreover, they reflect broader systemic issues in RACF settings. Further research should examine the decision-making processes in this setting.

### 4.4. Protocols, Policies, and Risk Culture

RACF policies, local and state-wide protocols, and a risk-averse culture contribute to transferring RACF residents to the ED after a fall. One concern regarding falls is intracranial bleeding in patients taking anticoagulants or antiplatelet agents [25]. Transferring post-fall patients to the ED despite no apparent injuries suggests a risk-avoiding behaviour [26].

However, protocol-driven post-fall monitoring increases the workload of RACF staff, leading to decisions to transfer patients to the ED [27]. In Australia, state policies, such as those of Victoria and Queensland, mandate rigorous post-fall observation, requiring up to 20 checks in 48 h [28,29]. While intended to ensure patient safety, such policies may overwhelm the already-strained RACF workforce. When RACF staff member resources are limited, transferring post-fall patients to the ED becomes the default option. Such decisions may be typically driven by the inability to meet mandated standards rather than the patient’s clinical deterioration.

Particularly in rural regions, where access to prompt primary care or medical imaging can often be limited, and RACF staff capacity is already constrained, such transfers to the ED post-fall may be the only feasible option [30]. However, this practice burdens healthcare systems and exposes residents to unnecessary disruption and stress, leading to poor adherence to patient-centred care [5,31].

Risk-averse cultures and clinical guidance also necessitate transferring patients to the ED after a fall, particularly those on anticoagulants, regardless of symptomatology [26]. Similarly to the existing literature, using anticoagulants or antiplatelets and the results of CTB scans were not significantly associated with ED disposition [26,32]. This indicates that routine CTB scans may offer few diagnostic benefits for post-fall patients [32]. They may reassure clinicians, but may also contribute to unnecessary ED presentations [33].

In addition, this study and previous reviews have demonstrated that CTB scans rarely reveal actionable findings in this cohort [26]. In this context, protocols designed to mitigate risk may lead to delivering low-value care, contrary to the patient-centred care in RACF settings. Policy reform should explicitly balance risk mitigation and prioritise patient-centred care.

## 5. Strengths and Limitations

This study provides novel insights into the characteristics of RACF residents discharged from the ED after a fall, an understudied yet clinically meaningful cohort with high healthcare utilisation. The analysis of numerous patient-level factors, including injury severity, the presence of obvious injuries, and ACD documentation, supports the development of targeted patient-centred strategies to reduce unnecessary ED transfers. Furthermore, the detailed analysis of ACDs provides excellent insights into areas that can improve patient-centred care.

However, this study has some limitations. First, because this study was conducted in a single-centred rural hospital, the generalisability of the study outcomes may be limited to local or rural populations. Second, the assumption of missing ACD data may have led to an underestimation of the prevalence of ACDs in this study. Third, this study did not review local RACF protocols influencing ED transfer decisions, thus limiting insights into facility-level decision-making. Fourth, we could not determine causation because this was an observational study. For example, while the presence of an ACD was correlated with ED discharge, residents with ACDs may have had other care preferences or unmeasured factors contributing to their less-frequent admissions. Other unmeasured factors such as frailty, cognition, and comorbidities may have influenced discharge decisions, thus introducing residual confounding. In addition, discharged patients were not followed-up with regarding outcomes. Further studies should examine the outcomes of discharged patients to ensure patient safety.

## 6. Conclusions

Among RACF residents who presented to the ED for a fall, major injuries and central body injuries warrant hospital admission. In contrast, the presence of an ACD and lower triage levels are associated with being discharged from the ED. Our findings suggest that the presence of ACDs—especially when accessible and specific—may help prevent unnecessary ED transfers. However, the lack of fall-related guidance within ACDs remains a key barrier to implementing patient-centred care. These findings highlight the need for policy initiatives that strengthen advance care planning in RACFs and emphasise the inclusion of fall-specific instructions in ACDs. It is also vital to enhance the capacity of RACFs—through staff training, communication with family members, and the revision of protocols—to reduce avoidable ED presentations and hospital admissions among older adults.

## Figures and Tables

**Table 1:** Characteristics of ED presentations following falls in RACFs by discharge status (n = 261).

Characteristics of ED Presentations				
	Total	Discharged	Admitted	*p* Value
	**261**	**181 (69.4%)**	**80 (30.6%)**	
**Age, median (max-min)**	87 (66–101)	87 (68–101)	86 (66–101)	0.538 ^†^
**Prior medical review**	50	37 (74.0%)	13 (26.0%)	0.428
**First Nations status**	9	6 (66.7%)	3 (33.3%)	0.819
**Duration of ED stay (mean minutes, SD)**	629.8 (450.7)	485.1 (368.2)	956.9 (450.9)	**<0.001**
**Use of anticoagulant or antiplatelet agents**	156	107 (68.6%)	49 (31.4%)	0.746
**Female**	166	115 (69.3%)	51 (30.7%)	0.974
**Means of Presentation, N (%)**				
**Ambulance**	250	172 (68.8%)	78 (31.2%)	0.359
**Private vehicle**	11	9 (81.82%)	2 (18.18%)	
**ATS, N (%)**				
**ATS-1 Immediate**	1	1 (100%)	0 (0%)	
**ATS-2 Time-Critical**	17	8 (47.1%)	9 (52.9%)	**0.028**
**ATS-3 Urgent**	216	149 (69.0%)	67 (31.0%)	
**ATS-4 Potential**	27	23 (85.2%)	4 (14.8%)	
**Injury Severity, N (%)**				
**Major**	132	73 (55.3%)	59 (44.7%)	**<0.001**
**Minor**	129	108 (83.7%)	21(16.3%)	
**Body Injury Region, N (%)**				
**Central**	180	48 (26.7%)	132 (73.3%)	**<0.001**
**Peripheral**	81	49 (60.5%)	32 (39.5%)	
**ACD, N (%)**				
**Presence of ACD**	115	91 (79.1%)	24 (20.9%)	**<0.001**
**Advised transfer to ED**	53	9 (17.0%)	44 (83.0%)	0.719 ^‡^
**Against transfer to ED**	15	3 (20.0%)	12 (80.0%)	
**ED Triage Notes, N (%)**				
**Fall**	257	177 (68.9%)	80 (31.1%)	0.316 ^‡^
**Obvious injuries**	140	85 (60.7%)	55 (39.3%)	**<0.001**
**Head strike**	71	52 (73.2%)	19 (26.8%)	0.405
**CTB, N (%)**				
**Had CTB**	192	138 (71.9%)	54 (28.1%)	0.140
**Normal CTB results**	180	133 (73.9%)	47 (26.1%)	**<0.001** ^‡^
**Abnormal CTB results**	11	4 (36.4%)	7 (63.6%)	

Bold text in the *p* values indicates *p* < 0.05. ^†^ Mann–Whitney U-test; ^‡^ Fisher’s exact test. All other p values were calculated using the chi-square test. *p* < 0.05 was considered statistically significant. Abbreviations: ACD = Advance Care Directive; CTB = computerised tomography brain scan; ATS = Australasian Triage Scale.

## Data Availability

Data are available upon reasonable request to the corresponding author.

## Appendix A. Inclusion Criteria

The inclusion criteria were based on the following:

1. ICD-10 with a fall-related presentation in either primary or secondary diagnosis.
  - W00-W19: Falls.
  - R29.6: Tendency to fall, not elsewhere classified.
  - X59: Exposure to unspecified factor causing other and unspecified injury.
  - R26: Abnormalities of gait and mobility.
  - R42: Dizziness and giddiness.
  - H81: Disorders of vestibular function.
  - R55: Syncope and collapse.
  - W25: Contact with sharp glass fall involving glass.
2. If, based on documentation from the ED notes, the injury’s cause was due to a fall.

## Appendix B. Injury Severity

Injury severity (major vs. minor):

Currently, the incident severity rating (ISR) from the Adverse Patient Safety Event guidelines from Safe Care Victoria is used to categorise injury severity. The ISR 1-4 has four levels: ISR 1—Severe: fractures, subdural haematomas, other major head trauma, cardiac arrest, and death; ISR 2—Moderate: excessive bleeding, lacerations requiring sutures, temporary loss of consciousness, moderate head trauma; ISR 3—Minor: minor cuts, minor bleeding, skin abrasions, swelling, pain, minor contusions; and ISR 4—No injury.

Based on the existing literature and the ISR from Adverse Patient Safety Event guidelines, injuries were classified into two categories as documented in the ED notes to stratify the level of injuries. If the patient had multiple minor injuries, they were classified as major; if the patient had major and minor injuries, they were classified as major.

Major injuries:

- Fracture;
- Intracranial injury;
- Multiple injuries;
- Dislocation;
- Crushing injury.

Minor injuries:

- Superficial;
- Laceration;
- Muscle/tendon injury;
- Sprain/strain;
- Open wound.

## Appendix C. Body Injury Location

Body injury location (central vs. peripheral):

Based on the criteria below and anatomical location, the body injury locations were classified as central or peripheral. Patients with central and peripheral injuries were classified as having a central injury.

Central classification:

- Hip;
- Back, lower (includes loin);
- Face;
- Neck;
- Abdomen;
- Pelvis (includes anogenital, perineum);
- Thorax;
- Head.

Peripheral body region:

- Knee;
- Ankle;
- Leg, lower;
- Shoulder;
- Forearm;
- Hand (includes fingers);
- Elbow;
- Foot (includes toes);
- Wrist;
- Thigh;
- Upper arm.

## Appendix D. STROBE Statement

**SECTION**	**ITEM NO.**	**RECOMMENDATION**	**PAGE NO.**
TITLE AND ABSTRACT	1	(a) Indicate the study’s design with a commonly used term in the title or the abstract	1
		(b) Provide in the abstract an informative and balanced summary of what was done and what was found	1
INTRODUCTION	2	Explain the scientific background and rationale for the investigation being reported	2
	3	State specific objectives, including any prespecified hypotheses	2
METHODS	4	Present key elements of study design early in the paper	2
	5	Describe the setting, locations, and relevant dates, including periods of recruitment, exposure, follow-up, and data collection	2
	6	Describe eligibility criteria and methods of selection of participants. Include rationale for design (cohort/case–control/cross-sectional) and follow-up	2
	7	Clearly define all outcomes, exposures, predictors, potential confounders, and effect modifiers; give diagnostic criteria, if applicable	3
	8	For each variable of interest, provide data sources and assessment methods; describe comparability if more than one group	2
	9	Describe any efforts to address potential sources of bias	3/4
	10	Explain how the study size was determined	4
	11	Explain handling of quantitative variables, including grouping decisions and rationale	3/4
	12	(a) Describe all statistical methods, including those used to control for confounding	3/4
		(b) Describe methods used to examine subgroups and interactions	3/4
		(c) Explain how missing data were addressed	4
		(d) Address loss to follow-up (cohort), matching (case–control), or sampling strategy (cross-sectional)	n/a
		(e) Describe any sensitivity analyses	4
RESULTS	13	(a) Report number of individuals at each stage (eligible, enrolled, analysed)	4
		(b) Give reasons for non-participation	n/a
		(c) Consider use of a flow diagram	5
	14	(a) Provide characteristics of participants and exposures/confounders	5/6
		(b) Indicate number of participants with missing data for each variable	n/a
		(c) Summarise follow-up time (if applicable)	n/a
	15	Report outcome events or summary measures (cross-sectional: outcomes; cohort: events over time; case–control: exposures)	6
	16	(a) Provide unadjusted and adjusted estimates with precision; state confounders adjusted for and rationale	6–8
		(b) Report category boundaries for categorised continuous variables	6–8
		(c) Translate relative risk into absolute risk if meaningful	n/a
	17	Report additional analyses (e.g., subgroups, interactions, sensitivity)	7–8
DISCUSSION	18	Summarise key results with reference to study objectives	8
	19	Discuss study limitations, including potential bias or imprecision, and their magnitude and direction	10
	20	Provide cautious interpretation considering limitations, multiple analyses, related studies, and other evidence	11
	21	Discuss generalisability (external validity)	9–10
OTHER INFORMATION	22	Disclose funding sources and roles of funders	11
